# Supplementary material for: Attempting to Create a Pathway to 15-Deacetylcalonectrin with Limited Accumulation in Cultures of Fusarium Tri3 Mutants: Insight into Trichothecene Biosynthesis Machinery
Source: Int J Mol Sci. 2024 Jun 11;25(12):6414. doi: 10.3390/ijms25126414 (PMC11203908; doi:10.3390/ijms25126414)
Supplement: Supplementary file 1 [file ijms-25-06414-s001.zip › ijms-3015079-supplementary.pdf]

Supplementary Materials

Brief Report

Attempting to Create a Pathway to 15-Deacetylcalonectrin with Limited Accumulation in Cultures of *Fusarium Tri3* Mutants: Insight into the Trichothecene Biosynthesis Machinery

Ena Kasahara[1,†], Yuna Kitamura[1,†], Miho Katada[1,†], Masashi Mizuki[1,†], Natsuki Okumura[1], Tomomi Sano[1], Yoshiaki Koizumi[2], Kazuyuki Maeda[1], Naoko Takahashi-Ando[2], Makoto Kimura[1,*], Yuichi Nakajima[1,*]

[1]*Department of Applied Biosciences, Graduate School of Bioagricultural Sciences, Nagoya University, Furo-cho, Chikusa-ku, Nagoya, Aichi 464-8601, Japan*
[2]*Graduate School of Science and Engineering, Toyo University, 2100 Kujirai, Kawagoe 350-8585 Saitama, Japan*

[†]joint first author

[*] Corresponding Authors: Makoto Kimura: mkimura@agr.nagoya-u.ac.jp; Yuichi Nakajima: ynakajima.gm@gmail.com

Supplementary Table S1. *Fusarium* strains and accession numbers of *EF-1α* and *RPB2* sequence used in molecular phylogenetic analysis.

Species	Strain[a]	GenBank accession nos.		References
		EF-1α	*RPB2*	
F. commune	MT-25	LC749603	LC749604	This study
F. beomiforme	RBG988	HQ667158	HQ646397	[1]
	RBG4549	HQ667157	HQ646396	[1]
F. commune	MRC 2564	MH582349	MH582180	[2]
	MRC 2566	MH582348	MH582181	[2]
	NRRL 28387	HM057338	JX171638	[2]
F. fujikuroi	MRC 2322	MH582343	MH582149	[2]
	MRC 2387	MH582340	MH582157	[2]
	MRC 2388	MH582341	MH582158	[2]
	MRC 2390	MH582342	MH582159	[2]
F. oxysporum	MRC 1694	MH582350	MH582094	[2]
	MRC 2066	MH582352	MH582126	[2]
	MRC 2199	MH582353	MH582140	[2]
	MRC 2325	MH582351	MH582152	[2]
	MRC 2536	MH582354	MH582173	[2]
F. proliferatum	MRC 2324	MH582344	MH582151	[2]
	MRC 2535	MH582346	MH582172	[2]
F. subglutinans	MRC 2627	MH582310	MH582187	[2]
	NRRL 22016	HM057336	JX171599	[3]
F. verticillioides	MRC 137	MH582315	MH582078	[2]
	NRRL 22172	MW402146	EF470122	[4]
				[5]

[a]RBG, Royal Botanic Gardens Trust, Sydney, New South Wales, Australia; MRC, South African Medical Research Council, Cape Town, South Africa; NRRL, The Agriculture Research Service Culture Collection, National Center for Agricultural Utilization Research, USDA/ARS, Peoria, IL, USA

(A)

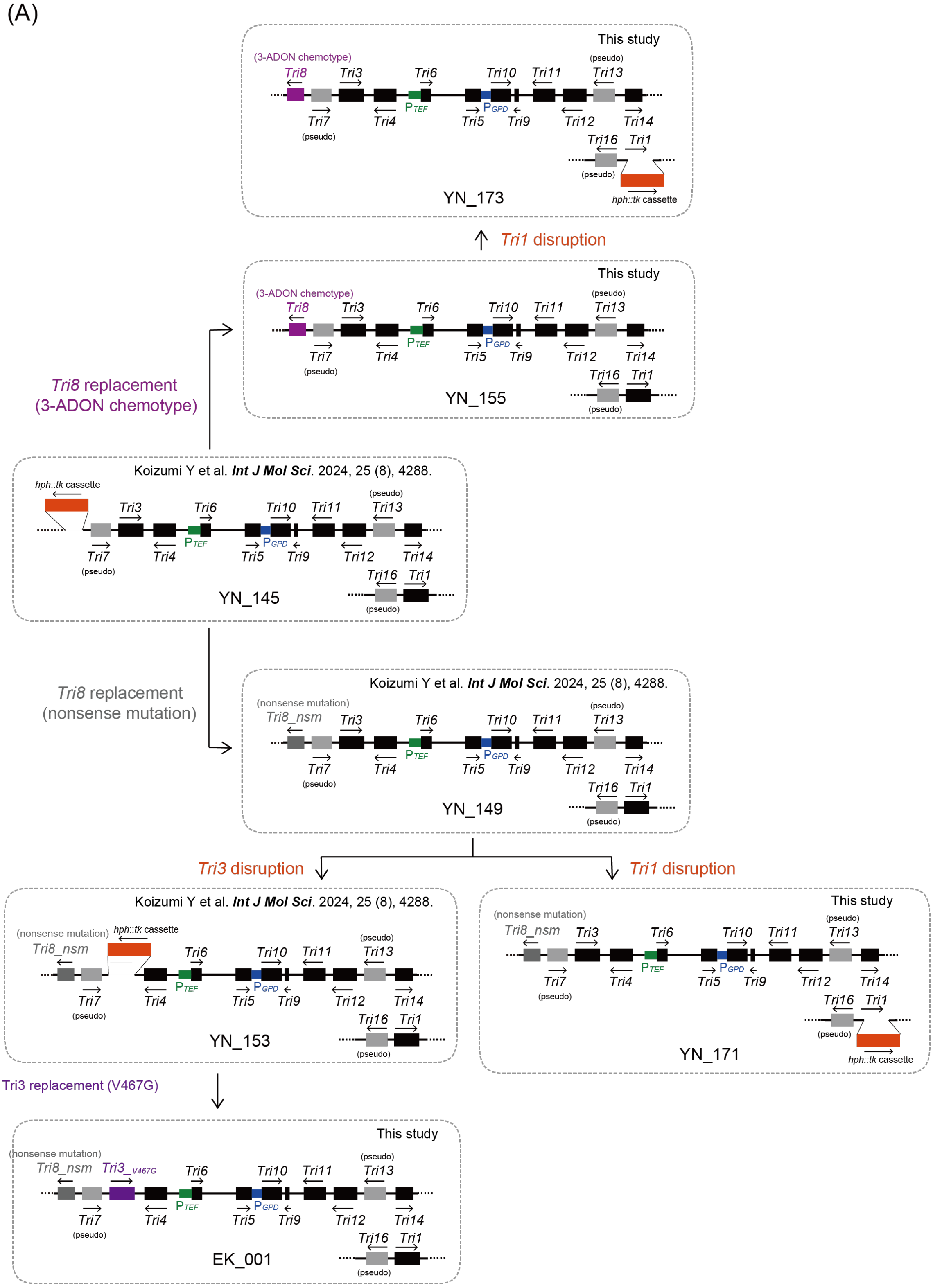
This study
(3-ADON chemotype)
YN_173
hph::tk cassette
Tri1 disruption
YN_155
Tri8 replacement
(3-ADON chemotype)
Koizumi Y et al. Int J Mol Sci. 2024, 25 (8), 4288.
YN_145
Tri8 replacement
(nonsense mutation)
YN_149
Tri3 disruption
Tri1 disruption
YN_153
YN_171
Tri3 replacement (V467G)
EK_001
Tri8
Tri8_nsm
Tri3_V467G
Tri7
Tri3
Tri4
Tri6
Tri5
Tri9
Tri10
Tri11
Tri12
Tri13
Tri14
Tri16
Tri1
(pseudo)
(nonsense mutation)
PTEF
PGPD

(B)

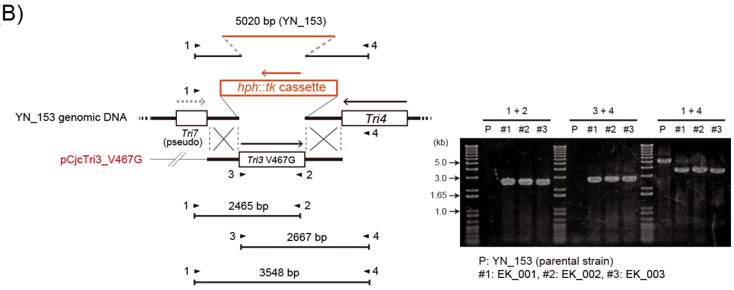

1 ► dTri3-Long_S: GAGCTTTGTGAACACCATTGGCGTCGACAC
2 ► GA_sw_Tri3_AS: AGAATAAACAGCAAATTACAGTTTGAATGCCAACATGAAC
3 ► GA_sw_Tri3_S: CCTACAACCATCAAGATGAGCGCTTCATCCTCCGCCTTGC
4 ► dTri3-Long_AS: GCGAGCTTACGACATTGAGCTTTATGACACC

↓

EK_001, EK_002, EK_003

(C)

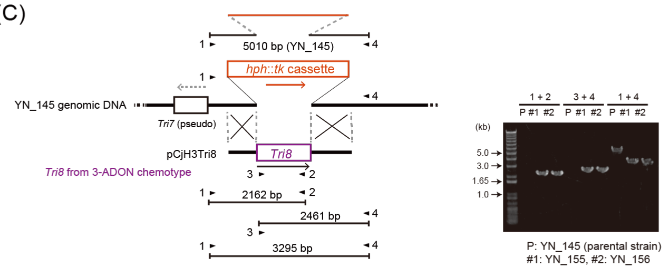

1 ► dTri8-Long_S: AGGACAATGACAGGAGAGGCATATCGCACC
2 ► F15-8sw-8_AS: GAAATGAAATCACAACTCTCGTGTTACGTCTCTC
3 ► F15-8sw-8_S: CACTCAGTATGGTTCTCGATCGTTTGTTGTTTCTTC
4 ► dTri8-Long_AS: GCTACATATACAGTAGGTATTTCTTCCTCCTGC

↓

YN_155, YN_156

(D)

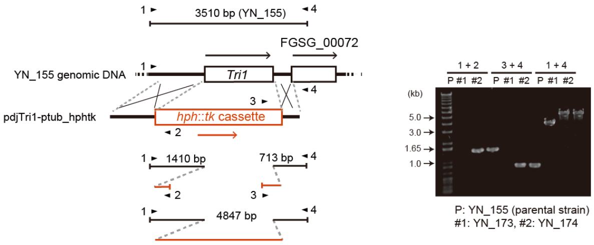

1 ► dTri1-Long_S: CCCGAGTAGACATCATGCCAACAGTTAGTTCTC
2 ► Specific_on_Ptub2: AACTGTGTAACTGCGTTATCGGTAGTGCGCTCC
3 ► Specific_on_HSVtk2: GCACGTCTTTATCCTGGATTACGACCAATCG
4 ► dTri1-Long_AS: CCGCCACATTCGCTAGTTTGTACTTAAGCGAG

↓

YN_173, YN_174

(E)

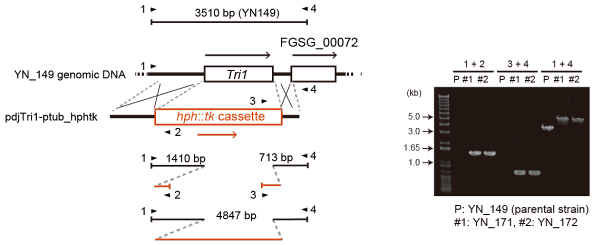

1 ► dTri1-Long_S: CCCGAGTAGACATCATGCCAACAGTTAGTTCTC
2 ► Specific_on_Ptub2: AACTGTGTAACTGCGTTATCGGTAGTGCGCTCC
3 ► Specific_on_HSVtk2: GCACGTCTTTATCCTGGATTACGACCAATCG
4 ► dTri1-Long_AS: CCGCCACATTCGCTAGTTTGTACTTAAGCGAG

↓

YN_171, YN_172

Supplementary Figure S1. Transgenic *F. graminearum* strains used in this study. Sequences and maps of transformation vectors are described in **Supplementary Figure S7**. (A) Pedigree of transformants with multiple genetic manipulations for production of trichothecene metabolites. Strain YN_145 (Δ*Tri6* / P_{TEF}::*Tri6*, ΔP_{Tri10} / P_{GPD}, Δ*Tri8* / P_{GPD}::*hph*::*tk*) is a *Tri8* disruptant derived from YN_120 (Δ*Tri6* / P_{TEF}::*Tri6*, ΔP_{Tri10} / P_{GPD}) [6]. Marker-free transformants YN_149 [6] and YN_155 (Δ*Tri6* / P_{TEF}::*Tri6*, ΔP_{Tri10} / P_{GPD}, Δ*Tri8* / *Tri8*$_{\_3\text{-}ADON\ chemotype}$) were obtained fromYN_145 by conditional negative selection against the *hph*::*tk* cassette with 2′-deoxy-5-fluorouridine (5-FdU). The gene disruptants, YN_171 (Δ*Tri6*/P_{TEF}::*Tri6*, ΔP_{Tri10}/P_{GPD}, Δ*Tri8*/*Tri8_nsm*, Δ*Tri1*/ P_{TUB}::*hph*::*tk*) and YN_173 (Δ*Tri6*/P_{TEF}::*Tri6*, ΔP_{Tri10}/P_{GPD}, Δ*Tri8*/*Tri8*$_{\_3\text{-}ADON\ chemotype}$, Δ*Tri1*/$P_{TUB}$::*hph*::*tk*), were obtained from marker-free transformants by a one-step transformation process, where hygromycin B resistant transformants with double crossover homologous recombination were selected against a marker cassette containing *hph*::*tk*, a hygromycin B phosphotransferase gene (*hph*) translationally fused to a herpes simplex virus thymidine kinase gene (*tk*). A marker-free transformant EK_001, EK_002, and EK_003 (Δ*Tri6* / P_{TEF}::*Tri6*, ΔP_{Tri10} / P_{GPD}, Δ*Tri3* / *Tri3*$_{\_V467G}$) was obtained from YN_153 [6], *Tri3* disruptant derived from YN_149. (B) Generation of EK_001, EK_002, and EK_003. EK_001, EK_002, EK_003 were created from YN_153 by selection with 5-FdU following transformation with pCjcTri3_V467G (**Supplementary Figure S7**) (C) Generation of strain YN_155. YN155 was created from YN_145 by selection with 5-FdU following transformation with pCjH3Tri8 (**Supplementary Figure S7**). (D) Generation of strain YN_173. YN173 was created from YN_155 by selection with hygromycin B following transformation with pdjTri1-ptub_hphtk (**Supplementary Figure S7**). (E) Generation of strain YN_171. YN_171 was created from YN_149 by selection with hygromycin B following transformation with pdjTri1-ptub_hphtk.

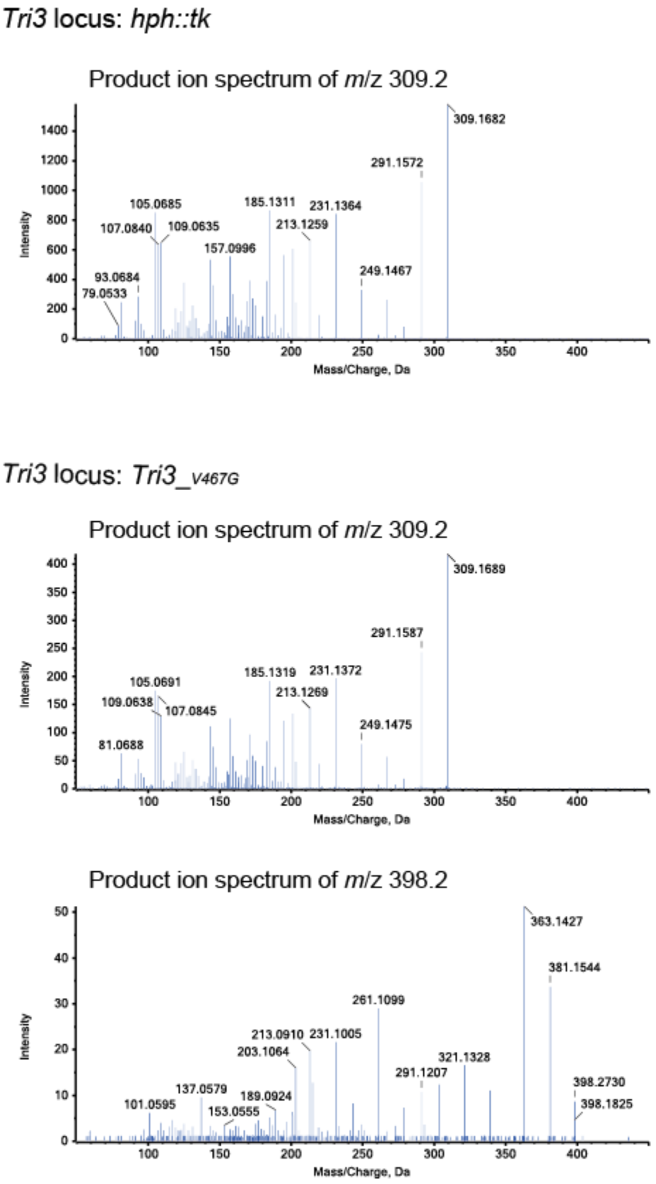

Supplementary Figure S2. LC-MS/MS analyses of the metabolites of the *Tri3* mutants (derived from a 3,15-diADON-overproducer described in **Figure 2**; native *Tri3* locus replaced with either the *hph::tk* maker or active-site mutant *Tri3*_$_{V467G}$) cultured on liquid YS_60 medium. The MS/MS spectra of the LC-MS peaks of *m*/*z* 309.2 (*hph::tk* and *Tri3*_$_{V467G}$) and *m*/*z* 398.2 (*Tri3*_$_{V467G}$) detected in the positive ion mode are shown. The precursor ions of *m*/*z* 309.2 and *m*/*z* 398.2 correspond to 15-deCAL [$C_{17}H_{24}O_5$ + H]$^+$ (*m*/*z* 309.1696) and 3,15-diADON [$C_{19}H_{24}O_8$ + NH_4]$^+$ (*m*/*z* 398.1809), respectively. Although either mutant produced 15-deCAL, the production level was extremely low compared to that of 3,15-diADON in the parent YN_149 strain.

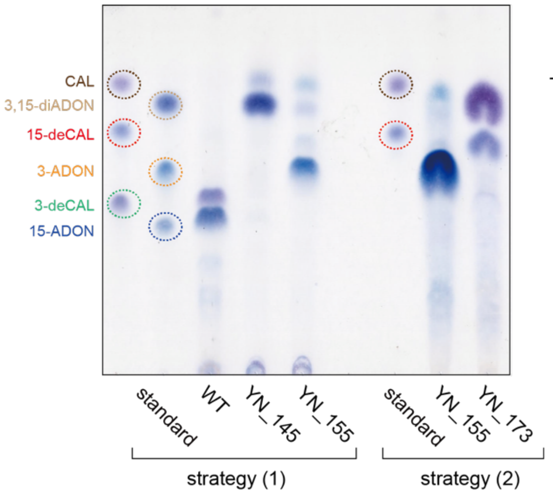

Transgenic strains derived from a trichothecene overproducer strain YN_120 (Δ*Tri6*/P$_{TEF}$::*Tri6*, ΔP$_{Tri10}$/P$_{GPD}$)

strain	*Tri8* locus	*Tri1* locus
YN_145	*hph::tk*	*Tri1* (native)
YN_155	*Tri8*_$_{3\text{-}ADON\ chemotype}$	*Tri1* (native)
YN_173	*Tri8*_$_{3\text{-}ADON\ chemotype}$	*hph::tk*

Supplementary Figure S3. TLC of culture metabolites of the transgenic *F. graminearum* strains. The three transgenic strains used in this experiment are derived from a 15-ADON overproducer strain YN_120 [6]. A synthetic methionine medium [7] and YG medium were used for culturing of the strain for strategy (1) and strategy (2), respectively. Ethyl acetate extract from 0.5 mL of the culture was loaded on each lane. After developing the TLC plate（Kieselgel F_{254}, Merck）with solvent mixture of ethyl acetate/toluene (3:1), trichothecenes were visualized with 4-(*p*-nitrobenzyl)pyridine (NBP)/tetraethylene pentamine (TEPA) [8].

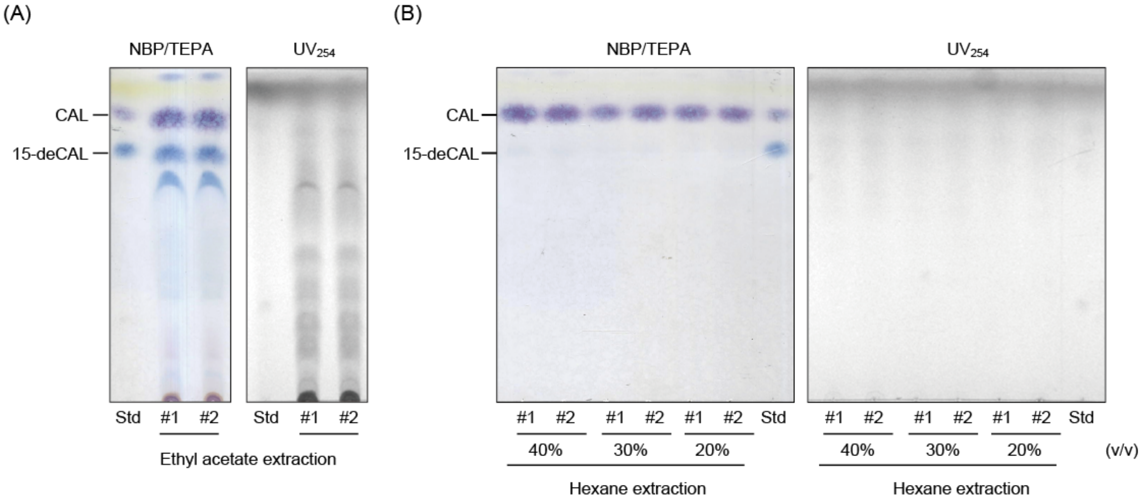

Supplementary Figure S4. Evaluation of the efficiency of the CAL extraction method. Each lane of TLC contains the metabolites extracted from 250 µL of the culture. To check the presence of fungal metabolites other than trichothecenes, the TLC plate was placed under UV light at 254 nm. The experiments were carried out in duplicate (#1 and #2). (A) A failure to directly use spent medium of the CAL-overproducer strain YN_171 (**Supplementary Figure S1**) for bioconversion of CAL to 15-deCAL. The conidia of YN_171 were inoculated onto YG medium at a final density of 1.0 × 10^4/mL and incubated for 16 h. One milliliter of the YG pre-culture thus prepared was transferred to 100 mL of YS_60 medium. After 6 days of incubation with gyratory shaking at 135 rpm and 25 °C, the CAL-containing spent medium was collected by filtration through a sterilized tea strainer (mesh size; 20 µm). The CAL-containing spent medium was aseptically supplemented with the nutrients of the synthetic 1 × NS medium (pH 4) and used for the feeding of strain MT-25. After 16 days of incubation, the whole culture was extracted with equal volume of ethyl acetate and analyzed by TLC. The TLC demonstrated the presence of CAL, its transformed product, and other fungal metabolites in the culture of strain MT-25; however, a significant amount of CAL still remained in the medium after 16 days of incubation. Besides, the presence of numerous contaminating metabolites, as demonstrated by the UV absorption, hampered efficient purification of the trichothecene metabolites. (B) Optimization of the CAL extraction method. By using the above spent medium containing CAL, its transformed product 15-deCAL, and other fungal metabolites, conditions for selective and efficient CAL extraction were investigated. When the culture was extracted with varying

amounts of hexane, the contaminating metabolites remained in the liquid phase; only CAL was detected from the solvent phase by the TLC analysis. The amounts of hexane used for the extraction are shown as percentage relative to the volume of the whole culture. By comparing the intensity of the CAL spots on a TLC plate, 40% the total culture volume of hexane proved to be sufficient in terms of quantity and quality for use in the subsequent feeding to *F. commune* MT-25 without further purification.

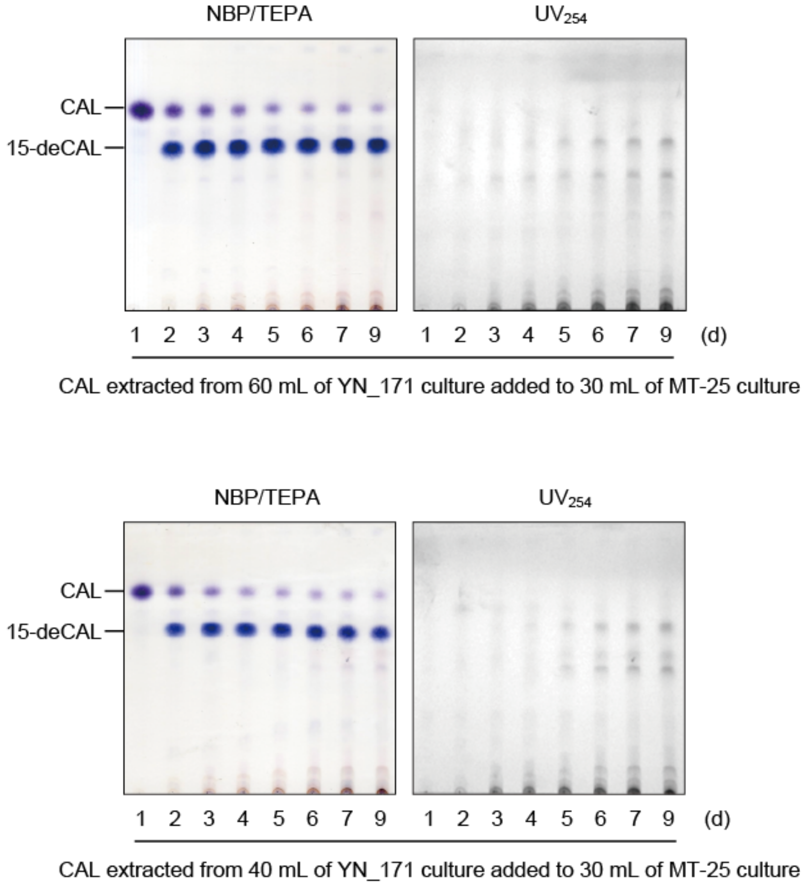

Supplementary Figure S5. Optimization of the amount of CAL and the period of incubation for bioconversion of CAL to 15-deCAL by MT-25. The CAL-overproducer YN_171 was grown on 100 mL of YS_60 medium and incubated for 6 days with gyratory shaking at 135 rpm and 25 °C. The whole culture was extracted with 40 mL of hexane, the solvent was concentrated in a hume hood, and the dried materials were dissolved in 50 µL of ethanol. Three-fifths (60 mL; upper panel) and two-fifths (40 mL; lower panel) the volumes of the CAL solutions were then added to 30 mL of fresh conidia of strain MT-25 (1.0×10^4/mL) dispersed in 1 × NS medium, pH 4. In adding the crude CAL extract to the MT-25 culture, the volume of ethanol was adjusted to 30 µL. Each culture was sampled on the day indicated, extracted with equal volume of ethyl acetate, and analyzed by TLC. Each lane contains metabolites extracted from 500 µL of the MT-25 culture.

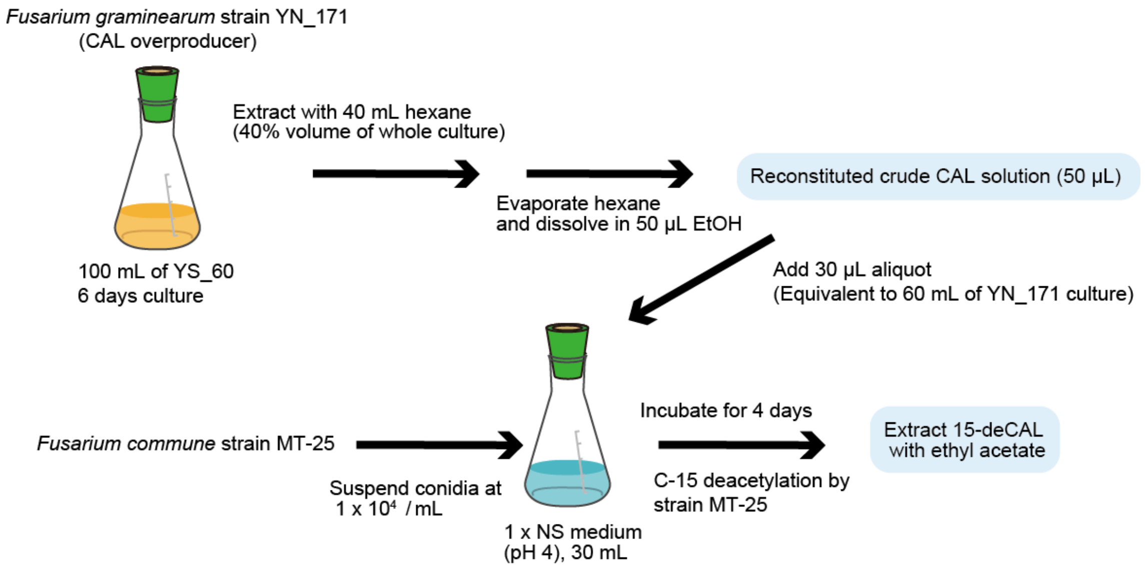

Supplementary Figure S6. An overview of the 15-deCAL preparation method established in this study. The extracted 15-deCAL can simply be purified in one step by preparative TLC or reversed phase column chromatography.

PstI Tri3_Upstream

1 GGGTAACGCC AGGGTTTTCC CAGTCACGAC GTTGTAAAAC GACGGCCAGT GCCAAGCTTG CATGCCTGCA GGTCGACTCT AGAGACCTTT TAAGC

Tri3_Upstream

101 ACGATATTGA GTTCGAATTT ATTAAGATAG ATACCATACA TAGATATCTG GTAATACAGA TGGTCATTGT CTCCATTATA AGAAGGCTGC ATAATA

Tri3_Upstream

201 TTTAACGAGA GTTTCGGCGA TCTGATGGAG TAAGCACGTG GGGCTACATG TGGATTCATC TGGCGCTTTG TATATTAAAT TATCTAAACC CGAGCA

Tri3_Upstream

301 GTTATACCTC TTTTCGTCGT AGGTTGCTTC ATGATACTGA GATACAAGAA TGATCCCTTC CCTGACTTGT GGTGCCGCTG GAAACTGAAG GAATGA

Tri3_Upstream

401 CCGACATTTA CAACAAACGT TGCATTTTCG CCATTGTGCC CTCCTAGATG TTTCGACTCA AGTCTGTAAC GTATTGAGAT GAACCAATGT GTGTCAC

Tri3_Upstream

501 ACTGAATCCT TGTCGAAAAA CTTGAATACA TGATGGAATG GCAAAAGTAG GTAAGAGATG CAAGGATAAG TGCTTGATCC GTCCGCTTAT GCCAG

Tri3_Upstream

601 CTAGACTAAG AATACGTCAA ACTTTGAACA AGTTAAGGCG TATCGTGTCA GTGGGGGTTG TGTAAGGCCC AACAGTGCAT TGTAAGCTAA GACTC

Tri3_Upstream

701 GCCTCAACTC CCTGTAAGTC TACAACATTT GTTGGCTCGG ATTGCGAAAC AAGGCTTCTA TTTATTAATA TAACTTGACA GCCAAGTATC TTCTGAG

GA_sw_Tri3_S

JCM Tri3 cDNA_V467G

Tri3_Upstream

+3 M S A S S S

801 TTCTAATAAC ACAGAGCTCT GATTTATCTT TGATTCTACA GACAAAAAAA AGCAAGCAAC TTATTTCATC CTACAACCAT CAAGATGAGC GCTTCAT

FgTri3-U_AS

JCM Tri3 cDNA_V467G

GA_sw_Tri3_S

+3 S A L P P L V P A L Y R W E S T G P R Q V Q R R C V G A E A I V G

901 CCGCCTTGCC ACCCTTGGTA CCAGCACTTT ACAGATGGGA GTCAACTGGT CCTCGTCAGG TCCAACGCCG GTGTGTCGGT GCTGAGGCAA TTGTT

JCM Tri3 cDNA_V467G

+3 L E E K N R R S L Y D L F I A T S L R N V A P A S T T L T L R N L K

1001 GGAAGAGAAG AACAGAAGAT CTCTATACGA TCTTTTCATC GCTACTTCTC TTCGGAATGT TGCACCAGCC TCAACAACGC TGACTTTGCG AAACCT

JCM Tri3 cDNA_V467G

+3 D M F E L A L V E G R F E H P E S A C T V S W D D Q V A A I I S Y

1101 GACATGTTCG AGCTGGCTTT GGTAGAGGGA CGTTTCGAGC ACCCCGAAAG TGCTTGTACA GTATCTTGGG ATGATCAAGT AGCTGCCATC ATCTC

JCM Tri3 cDNA_V467G

+3 E S P E S D E S V R D W A R G C V H V Q P T A K S A I D L W T E M

1201 AATCACCAGA GAGCGACGAA TCTGTTCGTG ATTGGGCTAG GGGTTGCGTT CATGTACAAC CTACCGCAAA GAGTGCTATT GACCTTTGGA CGGAG

JCM Tri3 cDNA_V467G

+3 E E G R A A A K D N T P S K P I E L F L L S D V P T D S T P I P Q G

1301 GGAAGGAAGA GCGGCAGCCA AGGATAACAC ACCATCCAAG CCGATTGAGC TCTTCCTGCT TTCAGATGTC CCTACAGACT CGACACCAAT CCCTC

JCM Tri3 cDNA_V467G

+3 A T V D I L F H S N H L F W D G I G C R K F I G D L F R L V G N H

1401 GCGACCGTCG ATATCTTGTT TCACAGCAAT CATTTGTTCT GGGACGGAAT CGGTTGTCGA AAGTTTATCG GGGACCTCTT TCGCCTCGTG GGCAAT

JCM Tri3 cDNA_V467G

+3 I G L S D S A E T H K I Q W G Q E I K N L S P P V V D S L K L D V

1501 TTGGCCTTAG CGACAGCGCT GAGACGCACA AGATACAATG GGGCCAAGAG ATCAAGAACC TGAGCCCTCC AGTCGTCGAC TCGCTGAAGT TGGA

JCM Tri3 cDNA_V467G

+3 S T L G T E F D D K C T E Y T S A L V A N Y K S R G M K F R P G L A

1601 CACTCTTGGA ACTGAGTTCG ACGACAAGTG CACAGAATAT ACAAGTGCTC TTGTGGCTAA TTATAAAAGC CGAGGTATGA AATTTCGACC AGGGC

JCM Tri3 cDNA_V467G

+3 L P R C A I Y K L S A D D S I A I I K A V K T R L G P G Y T I S Q

1701 TTGCCTCGTT GTGCTATTTA CAAACTCAGT GCCGACGACT CCATTGCCAT AATCAAGGCT GTGAAGACTC GCCTTGGCCC TGGCTATACC ATCAGT

JCM Tri3 cDNA_V467G

+3 L T Q A A I I L A L L D H L K P T D L S D D E F F L S P T S V D G

1801 TGACCCAAGC TGCCATCATA CTCGCTCTGC TGGACCATCT CAAGCCTACT GATCTTTCAG ACGACGAATT CTTCTTATCA CCAACATCAG TAGATG

JCM Tri3 cDNA_V467G

+3 R K W L R E D I A S K Y Y A M C Q T A A V V R V E N L K S I A V S H

1901 CAAGTGGCTG CGTGAGGATA TAGCCAGCAA ATACTACGCC ATGTGTCAGA CTGCTGCTGT TGTTCGCGTC GAGAATCTGA AGTCTATTGC AGTGA

JCM Tri3 cDNA_V467G

+3 K D E K E I Q V K A L E K A C R D I K K C Y D Q W L G N P F L E A

2001 AAGGATGAAA AAGAGATTCA AGTCAAAGCT CTAGAGAAGG CTTGCAGGGA TATCAAGAAA TGTTACGATC AATGGCTTGG AAATCCGTTC CTGG

JCM Tri3 cDNA_V467G

+3 L G L R V H N F E A G Y L N A K P N P F E G T E A N P L F I S D G V

2101 TGGGCCTCAG AGTCCATAAC TTCGAGGCCG GCTATTTGAA CGCCAAACCA AATCCGTTCG AAGGGACAGA AGCAAACCCG TTGTTCATCA GTGA

JTri3-V467G_S

V467G

JCM Tri3 cDNA_V467G

mutation

+3 N E R F I P R E I K R T T T G E D V L S V E S I D F V G N Q S L P

2201 CAACGAACGC TTCATTCCGC GCGAAATCAA GCGAACTACA ACAGGCGAGG ACGTGTTGTC CGTTGAGAGC ATAGACTTTG TTGGGAACCA GTCTTTGCCA

JTri3-V467m_AS

JTri3-V467G_S

JCM Tri3 cDNA_V467G

+3 Y L A I R L D S W R D A S T L N I I Y N D A N Y N E E E V Q T Y L

2301 TATCTGGCAA TCCGACTGGA CAGTTGGAGA GATGCTTCCA CCCTCAACAT CATCTATAAT GATGCAAACT ACAACGAGGA AGAGGTACAG ACGT

Tri3_Downstream

FgTri3-U_S

JCM Tri3 cDNA_V467G

+3 K S I V E F M L A F K L *

2401 AGAGTATTGT AGAGTTCATG TTGGCATTCA AACTGTAATT TGCTGTTTAT TCTGAATGGT ACATAGGTTT CTCGAGGAGC GATATTGTCA AGGCAA

GA_sw_Tri3_AS

Tri3_Downstream

2501 TTGGGGTGGA CTTTGAGTTC GGTGGTACTC CAAGGACAGG AAGACATTAC ATATGAGCTA GAACTGGATG GGTAAATGTA GTTTGAATAT AAATG

Tri3_Downstream

2601 GGAAATTTGA TTAGAATTTA CCTATTTACA ATTCAATTTT TTACTTCCCA AACTATGACT TATGTGGCTT GATATTAGAT CTTGGACAGG CCAGAGC

Tri3_Downstream

2701 ACTTCTATAA TTACAACTGC CTCTAGTTTC CACCAGGCCA TTTTTACATG CAATGAGCTT CGTTATATGT ATCAGCGCTC GTAGTATCAG TTCTCAA

Tri3_Downstream

2801 CACAAAAATA AGAGTGAGGA AGTCCCAGGT GAACAGTTTT CTAGGTTCAG AAAAGAGTAT AGATACTAAT TTGGTGAAGA CACATAGTCA ACAG

Tri3_Downstream

2901 GCAGTTGACT TTCTATATGC TAAACTACAA GATGAAGCCC AGTCCGTCTT GATGGACAAT CGCGATGTTC AACTGTGAGG TTAATGGTTT ACATGT

Tri3_Downstream

3001 AACACAATTG ATTGTGCCCT TAAAGCTAAC ATGAGTGGCC TTCACAAGTA ACCTACATGT CCAAAGCAGT TGATGTATGT AAATAATACT TAAGCT

Tri3_Downstream

3101 CTAAGTAAAG ACATATTATT AGTAATGCAG AGAAGACTTC CACAAGGAGT TAGTTTGATA GAATGGTCAC AAGTGTACGG TCTGTTATTC GTCCC

Tri3_Downstream

3201 ACATTGCCTG AGATGAATAG TGTTTAAATT GACGAGCGTC TAAATAGGGT ACAATTTATT TTACGTTCAG ATGTCGCTAC TATGACCTTC CATCCT

Tri3_Downstream

3301 TACCAAAAAT GGACTGAACA AAAGGAACAG GATTCGATGC CGATACAGTG AGTTATCCAG TAAGATATTG AGACAGTTTG TCAGATGCGC CGGGGTACCG

3401 AGCTCGAATT CGTAATCATG GTCATAGCTG TTTCCTGTGT GAAATTGTTA TCCGCTCACA ATTCCACACA ACATACGAGC CGGAAGCATA AAGTGTAAAG

3501 CCTGGGGTGC CTAATGAGTG AGCTAACTCA CATTAATTGC GTTGCGCTCA CTGCCCGCTT TCCAGTCGGG AAACCTGTCG TGCCAGCTGC ATTAATGAAT

3601 CGGCCAACGC GCGGGGAGAG GCGGTTTGCG TATTGGGCGC TCTTCCGCTT CCTCGCTCAC TGACTCGCTG CGCTCGGTCG TTCGGCTGCG GCGAGCGGTA

3701 TCAGCTCACT CAAAGGCGGT AATACGGTTA TCCACAGAAT CAGGGGATAA CGCAGGAAAG AACATGTGAG CAAAAGGCCA GCAAAAGGCC AGGAACCGTA

3801 AAAAGGCCGC GTTGCTGGCG TTTTTCCATA GGCTCCGCCC CCCTGACGAG CATCACAAAA ATCGACGCTC AAGTCAGAGG TGGCGAAACC CGACAGGACT

3901 ATAAAGATAC CAGGCGTTTC CCCCTGGAAG CTCCCTCGTG CGCTCTCCTG TTCCGACCCT GCCGCTTACC GGATACCTGT CCGCCTTTCT CCCTTCGGGA

4001 AGCGTGGCGC TTTCTCATAG CTCACGCTGT AGGTATCTCA GTTCGGTGTA GGTCGTTCGC TCCAAGCTGG GCTGTGTGCA CGAACCCCCC GTTCAGCCCG

4101 ACCGCTGCGC CTTATCCGGT AACTATCGTC TTGAGTCCAA CCCGGTAAGA CACGACTTAT CGCCACTGGC AGCAGCCACT GGTAACAGGA TTAGCAGAGC

4201 GAGGTATGTA GGCGGTGCTA CAGAGTTCTT GAAGTGGTGG CCTAACTACG GCTACACTAG AAGGACAGTA TTTGGTATCT GCGCTCTGCT GAAGCCAGTT

4301 ACCTTCGGAA AAAGAGTTGG TAGCTCTTGA TCCGGCAAAC AAACCACCGC TGGTAGCGGT GGTTTTTTTG TTTGCAAGCA GCAGATTACG CGCAGAAAAA

4401 AAGGATCTCA AGAAGATCCT TTGATCTTTT CTACGGGGTC TGACGCTCAG TGGAACGAAA ACTCACGTTA AGGGATTTTG GTCATGAGAT TATCAAAAAG

4501 GATCTTCACC TAGATCCTTT TAAATTAAAA ATGAAGTTTT AAATCAATCT AAAGTATATA TGAGTAAACT TGGTCTGACA GTTACCAATG CTTAATCAGT

bla

4601 GAGGCACCTA TCTCAGCGAT CTGTCTATTT CGTTCATCCA TAGTTGCCTG ACTCCCCGTC GTGTAGATAA CTACGATACG GGAGGGCTTA CCATCTGGCC

bla

4701 CCAGTGCTGC AATGATACCG CGAGACCCAC GCTCACCGGC TCCAGATTTA TCAGCAATAA ACCAGCCAGC CGGAAGGGCC GAGCGCAGAA GTGGTCCTGC

bla

4801 AACTTTATCC GCCTCCATCC AGTCTATTAA TTGTTGCCGG GAAGCTAGAG TAAGTAGTTC GCCAGTTAAT AGTTTGCGCA ACGTTGTTGC CATTGCTACA

bla

4901 GGCATCGTGG TGTCACGCTC GTCGTTTGGT ATGGCTTCAT TCAGCTCCGG TTCCCAACGA TCAAGGCGAG TTACATGATC CCCCATGTTG TGCAAAAAAG

bla

5001 CGGTTAGCTC CTTCGGTCCT CCGATCGTTG TCAGAAGTAA GTTGGCCGCA GTGTTATCAC TCATGGTTAT GGCAGCACTG CATAATTCTC TTACTGTCAT

bla

5101 GCCATCCGTA AGATGCTTTT CTGTGACTGG TGAGTACTCA ACCAAGTCAT TCTGAGAATA GTGTATGCGG CGACCGAGTT GCTCTTGCCC GGCGTCAATA

bla

5201 CGGGATAATA CCGCGCCACA TAGCAGAACT TTAAAAGTGC TCATCATTGG AAAACGTTCT TCGGGGCGAA AACTCTCAAG GATCTTACCG CTGTTGAGAT

bla

5301	CCAGTTCGAT	GTAACCCACT	CGTGCACCCA	ACTGATCTTC	AGCATCTTTT	ACTTTCACCA	GCGTTTCTGG	GTGAGCAAAA	ACAGGAAGGC	AAAATC
	bla									
5401	AAAAAAGGGA	ATAAGGGCGA	CACGGAAATG	TTGAATACTC	ATACTCTTCC	TTTTTCAATA	TTATTGAAGC	ATTTATCAGG	GTTATTGTCT	CATGAGCGGA
	bla									
5501	TACATATTTG	AATGTATTTA	GAAAAATAAA	CAAATAGGGG	TTCCGCGCAC	ATTTCCCCGA	AAAGTGCCAC	CTGACGTCTA	AGAAACCATT	ATTATCATGA
5601	CATTAACCTA	TAAAAATAGG	CGTATCACGA	GGCCCTTTCG	TCTCGCGCGT	TTCGGTGATG	ACGGTGAAAA	CCTCTGACAC	ATGCAGCTCC	CGGAGACGGT
5701	CACAGCTTGT	CTGTAAGCGG	ATGCCGGGAG	CAGACAAGCC	CGTCAGGGCG	CGTCAGCGGG	TGTTGGCGGG	TGTCGGGGCT	GGCTTAACTA	TGCGGCATCA
5801	GAGCAGATTG	TACTGAGAGT	GCACCATATG	CGGTGTGAAA	TACCGCACAG	ATGCGTAAGG	AGAAAATACC	GCATCAGGCG	CCATTCGCCA	TTCAGGCTGC
5901	GCAACTGTTG	GGAAGGGCGA	TCGGTGCGGG	CCTCTTCGCT	ATTACGCCAG	CTGGCGAAAG	GGGGATGTGC	TGCAAGGCGA	TTAAGTT	

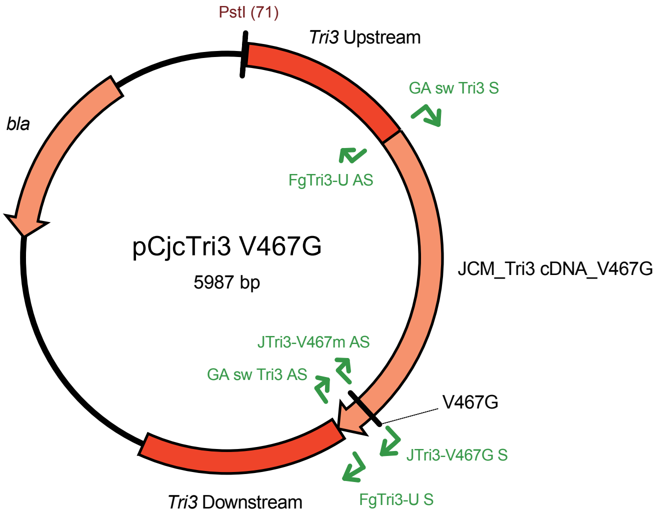

							PstI; dTri8-U_S	dTri8-U_S	dTri8-U_S; Tri8 upstream region	dTri8-U_S; Tri8 upstream region
1	GGGTAACGCC	AGGGTTTTCC	CAGTCACGAC	GTTGTAAAAC	GACGGCCAGT	GCCAAGCTTG	CATGCCTGCA	GGTCGACTCT	AGAGTCTACC	TGCC
	dTri8-U_S; Tri8 upstream region	Tri8 upstream region								
101	TACCTATCGC	ATTGTCGCTG	GTGCTGGAAA	AGATTCCGTC	CGACTTTCGG	TCCGATTAGA	ACTATTGCGA	AGGCCGTGAA	AATGAACCGC	TTGAT
	Tri8 upstream region									
201	AAGACTAGAG	GTCCAGACAA	GTAAGTGAAA	GAGCAATACA	AGACAGATGA	AGCCTTACTT	TTCTCAGGCC	TCGCTGGTGG	ACCTAGTAAA	AAG
	Tri8 upstream region									
301	TCGAAACGAA	CCAGCTGTGA	TGTGTAGAAT	GGAGGGCCTG	ATGAAAGGGC	CAGGGGCCTG	ACATGGACAT	GGGGACAATA	AATCGGCTGT	TC
	Tri8 upstream region									
401	CGTAACTACA	CTTAAAGAAC	CCAGGACTCA	ATCTAGAATT	CTTTGATAAA	GCGAGCTTCA	CTTTACTATG	TATAAAGTAA	AGTTTGTAGT	GTATAC

Tri8 upstream region

501 TTAGCCCTTC CTCATTTGAT TACAGTTCTA ATTCTTGTAA GACTTGGTGA TGGTTAGGAG CCCCCTGTAA GTTTGTGCGC TCCTATGGCT CAAAATC

Tri8 upstream region

601 CATTTCACTC TAGCGCATTG AGGATTGATG TACTCTGTAC GTGCTGAGGT CTTGGACCCC TTCACCCCTG GCACAACAAC ATCAAGGTTC AAACA

F15-8sw-8_S

Tri8_3-ADON chemotype

Tri8 upstream region

+3 M V L D

701 ACTAAATAAA ACGATGCGCC GCGTTTCGTA GTGTGAGTCC CAAAGAGCTA TTGACAGTTC AAGTCATTCA TTCGTTCACT CACAT GGTTCTCGAT

F15-8sw-U_AS

Tri8_3-ADON chemotype

F15-8sw-8_S

+3 R L L F L L S L W L G F V G A T Q A A L S E P I P P S K D P W Y T A·

801 CGTTTGTTGT TTCTTCTGAG CTTGTGGCTG GGCTTTGTTG GCGCCACTCA AGCTGCGTTA TCAGAACCTA TTCCGCCTAG CAAGGACCCG TGGTAT

Tri8_3-ADON chemotype

+3 ·A P L G F E N A E P G T V F R V R P A P G N L T S V I G N C S A S Y·

901 CACCTCTTGG ATTTGAGAAT GCCGAGCCTG GAACTGTCTT TAGAGTGCGG CCTGCGCCTG GAAACTTAAC CAGCGTCATC GGCAATTGCT CTGCT

Tri8_3-ADON chemotype

+3 ·Y N I L Y R T T D S Q F K P T W A V T T L L I P K L G P E S L A Q Q

1001 CAACATCCTC TACCGTACGA CCGACAGCCA GTTCAAGCCT ACTTGGGCCG TCACCACTCT TTTAATCCCC AAACTGGGCC CAGAAAGTCT TGCGC

Tri8_3-ADON chemotype

+3 K Y Q Q S A L M S F Q V P Y D S P D V D A S P S N A M Y D A S D F F·

1101 AAGTACCAAC AAAGCGCGCT CATGTCATTC CAAGTGCCTT ATGACTCCCC CGATGTCGAT GCCAGCCCTA GTAACGCTAT GTACGACGCG AGCG

Tri8_3-ADON chemotype

+3 ·F S N Y Y G A A L G E G I F V S V P D Y E G P L A A F T A G L I S G·

1201 TTTCGAATTA CTATGGAGCT GCTCTAGGTG AAGGTATCTT CGTTTCTGTT CCGGATTATG AGGGACCCTT GGCTGCTTTT ACCGCTGGTC TCATCTC

Tri8_3-ADON chemotype

+3 ·G Y A T L D S I R A V L S L G L G F N T I H T P S V A L W G Y S G G

1301 ATATGCTACT TTGGACTCGA TTCGTGCTGT TTTGTCGCTC GGTCTTGGTT TCAACACGAT CCACACGCCC AGTGTTGCCC TTTGGGGCTA TTCTGGC

Tri8_3-ADON chemotype

+3 A F A T E W A S E L A V Q Y A P E L V A G P V I G A V M G A P L P N·

1401 GCGTTTGCCA CCGAATGGGC ATCAGAACTT GCAGTACAGT ATGCACCCGA GCTTGTAGCA GGACCCGTTA TTGGTGCTGT GATGGGAGCT CCGT

Tri8_3-ADON chemotype

+3 ·N I T S C M R D V N G G P K S G L V V N M L L G L T S Q Y P D V R K·

1501 ATATCACTTC ATGCATGCGC GATGTCAATG GAGGACCTAA GTCCGGTCTG GTGGTAAACA TGCTGTTGGG TCTCACGAGT CAATATCCTG ATGTC

Tri8_3-ADON chemotype

+3 ·K H L V S K L N D D G Q Y N K A D F L A A E G F S I G K A L D T F S

1601 GCACCTTGTA TCCAAACTCA ACGATGATGG CCAGTACAAC AAGGCTGACT TTCTCGCTGC TGAGGGTTTT AGTATCGGCA AAGCGCTTGA CACCT

Tri8_3-ADON chemotype

+3 G N I N K Y F Q K G T D I L S D P K I T A L I N R E G V L G Y H G T·

1701 GGCAACATCA ACAAGTACTT CCAGAAGGGA ACTGATATTC TCAGCGACCC GAAGATTACA GCTCTTATCA ACCGGGAGGG CGTTTTGGGA TATC

Tri8_3-ADON chemotype

+3 ·T P R W P M F I Y Q A I A D E V T P I A A T D A V V E R Y C S V G A·

1801 CCCCCAGATG GCCCATGTTT ATTTACCAAG CTATCGCTGA CGAGGTCACG CCGATTGCTG CTACCGATGC CGTAGTCGAG AGATATTGTT CGGTC

Tri8_3-ADON chemotype

+3 ·A D V H F E R N T L G S H D E E A N N S Y E A A F Q W L L N I F S G

1901 CGACGTTCAC TTCGAAAGGA ACACTCTCGG CTCGCACGAC GAGGAAGCCA ACAATAGCTA TGAAGCGGCT TTCCAGTGGC TTTTGAACAT CTTC

F15-8sw-D_S

Tri8_3-ADON chemotype

Tri8 downstream region

+3 Q R D T K G C V I K D V T R D V T G D V T R D V T R D V T R E L *

2001 CAGCGTGACA CAAAGGGTTG TGTTATCAAG GACGTGACGA GAGACGTGAC GGGAGACGTG ACGAGAGACG TGACGAGAGA CGTAACACGA AT

F15-8sw-8_AS

F15-8sw-D_S

Tri8 downstream region

2101 TTCATTTCTT GACATCTCTG GCCATCGGCC TGGTAATTTA TTTTTGGACT GCCGGTCATC GACTTGGTAC GCCATCCCTT ATATGTTTGG CCAGAAC

F15-8sw-8_AS

Tri8 downstream region

2201 TAGTGGTCTT ATACTGTGTC TTATAAAATA TTTATTGATC CTTTTCTATG AGTAACTTTA TCTGTCTCCG GCGATGTAGC TGCATCTGTA AGATTCAA

Tri8 downstream region

2301 AAATCATCCA AACACCAGCC AGGTAGCATT AATCGAAGAG GACCTCCAAT CTGAATAACT TCGGTGTTGT TGAGTATTCC TGGTTTATTG AGATG

Tri8 downstream region

2401 GATCAGCTCC CATTTCCCTT CAAGTTCCCT CCATTGTGGT CAGAAGACAA GGGAAGCATG AGGGTGGACT CATTTGAAGC ACGATAATTA CACT

Tri8 downstream region

2501 AAACACAGGT TTAGCTTGAA CTGAAAAACC ACTCATCACA TTCACAGTAG CACCACTGGA TAGATGTTGG GTTCAAGTAA GCCTCATGTT GCTCT

Tri8 downstream region

2601 CGCTACTTAC CCCTTGCCGC AACAATATGC ATCTTTTACA CTTCATGCAC AAACAAGGGC ATGGTATCGT TGAGGCAATA TCAGACATAT AAATT

Tri8 downstream region

2701 TAATATTGTC GGATACTGCG TGGAGGAAGG CGACTTCGTC TTTATGGACG TCAAGCCCTC CTGTAAGCCT CTTTTCAAGG TCCCTCAGGC TCGCA

Tri8 downstream region

2801 CTTCTCAAGC TCTGGCACTA TCCGGCCTAG CTTCTTAGAT CGAAAGCGCC TTACGAAGAG CAAGAGGACC TTGTAGCTGA AGGCAATTAA CCTG

Tri8 downstream region

2901 CTGTAGTTAT CGTCATATGG CCCCTCTCGG AGGCACCTTA CCTTATGCCT TAGCTACTTT AGATCCTTAC TGTAGTTACT AAGTAAGATA ACCTGGT

Tri8 downstream region

3001 TGCCTTCTTT AGTAAAGAAA TAGTCTTCTA GATAGCTAGG GATATAACTG TATAGGCTTT TTTATTGAGG CTGTATTATT ATATAATACC TATGACA

Tri8 downstream region

3101 GTTAGCTATA TGGAGCGATG GACTGGGGTA CCGAGCTCGA ATTCGTAATC ATGGTCATAG CTGTTTCCTG TGTGAAATTG TTATCCGCTC ACAAT

dTri8-D_AS

3201 ACAACATACG AGCCGGAAGC ATAAAGTGTA AAGCCTGGGG TGCCTAATGA GTGAGCTAAC TCACATTAAT TGCGTTGCGC TCACTGCCCG CTTTCCAGTC

3301 GGGAAACCTG TCGTGCCAGC TGCATTAATG AATCGGCCAA CGCGCGGGGA GAGGCGGTTT GCGTATTGGG CGCTCTTCCG CTTCCTCGCT CACTGACTCG

3401 CTGCGCTCGG TCGTTCGGCT GCGGCGAGCG GTATCAGCTC ACTCAAAGGC GGTAATACGG TTATCCACAG AATCAGGGGA TAACGCAGGA AAGAACATGT

3501 GAGCAAAAGG CCAGCAAAAG GCCAGGAACC GTAAAAAGGC CGCGTTGCTG GCGTTTTTCC ATAGGCTCCG CCCCCCTGAC GAGCATCACA AAAATCGACG

3601 CTCAAGTCAG AGGTGGCGAA ACCCGACAGG ACTATAAAGA TACCAGGCGT TTCCCCCTGG AAGCTCCCTC GTGCGCTCTC CTGTTCCGAC CCTGCCGCTT

3701 ACCGGATACC TGTCCGCCTT TCTCCCTTCG GGAAGCGTGG CGCTTTCTCA TAGCTCACGC TGTAGGTATC TCAGTTCGGT GTAGGTCGTT CGCTCCAAGC

3801 TGGGCTGTGT GCACGAACCC CCCGTTCAGC CCGACCGCTG CGCCTTATCC GGTAACTATC GTCTTGAGTC CAACCCGGTA AGACACGACT TATCGCCACT

3901 GGCAGCAGCC ACTGGTAACA GGATTAGCAG AGCGAGGTAT GTAGGCGGTG CTACAGAGTT CTTGAAGTGG TGGCCTAACT ACGGCTACAC TAGAAGGACA

4001 GTATTTGGTA TCTGCGCTCT GCTGAAGCCA GTTACCTTCG GAAAAAGAGT TGGTAGCTCT TGATCCGGCA AACAAACCAC CGCTGGTAGC GGTGGTTTTT

4101 TTGTTTGCAA GCAGCAGATT ACGCGCAGAA AAAAAGGATC TCAAGAAGAT CCTTTGATCT TTTCTACGGG GTCTGACGCT CAGTGGAACG AAAACTCACG

4201 TTAAGGGATT TTGGTCATGA GATTATCAAA AAGGATCTTC ACCTAGATCC TTTTAAATTA AAAATGAAGT TTTAAATCAA TCTAAAGTAT ATATGAGTAA

4301 ACTTGGTCTG ACAGTTACCA ATGCTTAATC AGTGAGGCAC CTATCTCAGC GATCTGTCTA TTTCGTTCAT CCATAGTTGC CTGACTCCCC GTCGTGTAGA

bla

4401 TAACTACGAT ACGGGAGGGC TTACCATCTG GCCCCAGTGC TGCAATGATA CCGCGAGACC CACGCTCACC GGCTCCAGAT TTATCAGCAA TAAACCAGCC

bla

4501 AGCCGGAAGG GCCGAGCGCA GAAGTGGTCC TGCAACTTTA TCCGCCTCCA TCCAGTCTAT TAATTGTTGC CGGGAAGCTA GAGTAAGTAG TTCGCCAGTT

bla

4601 AATAGTTTGC GCAACGTTGT TGCCATTGCT ACAGGCATCG TGGTGTCACG CTCGTCGTTT GGTATGGCTT CATTCAGCTC CGGTTCCCAA CGATCAAGGC

bla

4701 GAGTTACATG ATCCCCCATG TTGTGCAAAA AAGCGGTTAG CTCCTTCGGT CCTCCGATCG TTGTCAGAAG TAAGTTGGCC GCAGTGTTAT CACTCATGGT

bla

4801 TATGGCAGCA CTGCATAATT CTCTTACTGT CATGCCATCC GTAAGATGCT TTTCTGTGAC TGGTGAGTAC TCAACCAAGT CATTCTGAGA ATAGTGTATG

bla

4901 CGGCGACCGA GTTGCTCTTG CCCGGCGTCA ATACGGGATA ATACCGCGCC ACATAGCAGA ACTTTAAAAG TGCTCATCAT TGGAAAACGT TCTTCGGGGC

bla

5001 GAAAACTCTC AAGGATCTTA CCGCTGTTGA GATCCAGTTC GATGTAACCC ACTCGTGCAC CCAACTGATC TTCAGCATCT TTTACTTTCA CCAGCGTTTC

bla

5101 TGGGTGAGCA AAAACAGGAA GGCAAAATGC CGCAAAAAAG GGAATAAGGG CGACACGGAA ATGTTGAATA CTCATACTCT TCCTTTTTCA ATATTATTGA

bla

5201 AGCATTTATC AGGGTTATTG TCTCATGAGC GGATACATAT TTGAATGTAT TTAGAAAAAT AAACAAATAG GGGTTCCGCG CACATTTCCC CGAAAAGTGC

5301 CACCTGACGT CTAAGAAACC ATTATTATCA TGACATTAAC CTATAAAAAT AGGCGTATCA CGAGGCCCTT TCGTCTCGCG CGTTTCGGTG ATGACGGTGA

5401 AAACCTCTGA CACATGCAGC TCCCGGAGAC GGTCACAGCT TGTCTGTAAG CGGATGCCGG GAGCAGACAA GCCCGTCAGG GCGCGTCAGC GG

5501 GGGTGTCGGG GCTGGCTTAA CTATGCGGCA TCAGAGCAGA TTGTACTGAG AGTGCACCAT ATGCGGTGTG AAATACCGCA CAGATGCGTA AGGAGAAAAT

5601 ACCGCATCAG GCGCCATTCG CCATTCAGGC TGCGCAACTG TTGGGAAGGG CGATCGGTGC GGGCCTCTTC GCTATTACGC CAGCTGGCGA AAGGGGGATG

5701 TGCTGCAAGG CGATTAAGTT

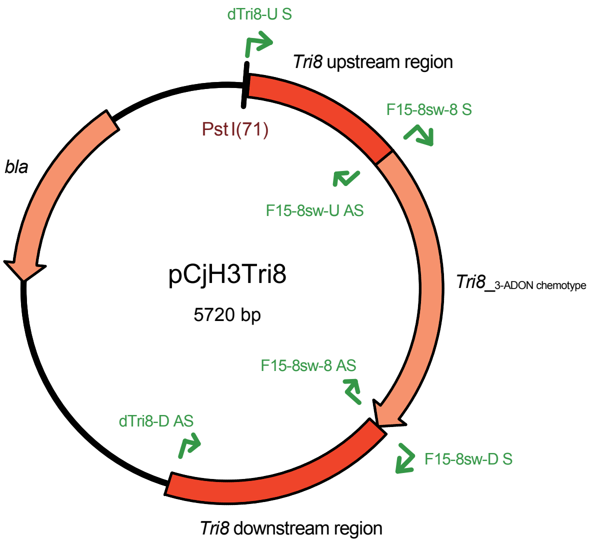

dTri1-U_S
HindIII
Tri1 upstream region

1 GGGTAACGCC AGGGTTTTCC CAGTCACGAC GTTGTAAAAC GACGGCCAGT GCCAAGCTTG CATGCCTGCA GGTCGACTCT AGAGGCCTCA TAG

dTri1-U_S
Tri1 upstream region

101 CTTGCCGTAC TGGTACACTG TTGCGAAATT AATATCCAAT GAGAAACCAA GTCAAGTCGG TCTATGAGTC TGTGGGGTGG ATGGCCTGAG AGTC

Tri1 upstream region

201 ACCAACATTG AAACACCAAA GTCAATTACA GGGCCTCTGC AGATACCCTA TAGAGAGGTT GGAGGCAGTA AATAAATAGA TGGAATGTTG AAG

Tri1 upstream region

301 AGATTACGGA TAATACATGT ATGGTCTCGT AGTATAATTG ATGAGAATTT AATAAATATA TTTCCTTGTT AAGAATTTAA TTATTTTGCT GTCCAAG

Tri1 upstream region

401 ACTTCATGAC AGCTTTTGCC ATTATGACTG CTTACAGTAA TCCAGAGCCT TTGCCTTACT CGGCAGAAAC TTGTGCATTG TTTCACAAAG CTAGAC

Tri1 upstream region

501 AGTAGTTGCC GATAGTTAGA CCTTTACAGT CCTCAAGACA AACAAACAGA CACCATTTGT AGTTTCAAGG AGTGGTCGAT GGTGCCCTTT TGTCT

Tri1 upstream region

601 AAGGTTACGT GTACTGGTAA ATTTGAAACC ACGGCTGAGT ATCGTAATGT CAAAGTCAAC CGCTGATGCC AGGACTGTGT GGTGAGTAAG TGGC

Tri1 upstream region

701 ACCTAGCAGG CTGGAAGTTA CGAATCTAGA CTAGCATGTG AAGATAACGA TACCAAAGTC AATTCTGTTT GGTTACAAGT TTTGGATCTT GTGCA

Tri1 upstream region

801 GCCTGATAGT TAGTACCCTA CCAATCAATA TGAAGATCAA TGCAACAAAG TCAGTATTAT TCGGTAGGTC GTTTTGTTCG TTAGTGGGGG TTGGC

Tri1 upstream region

901 TCTACCAATT GAGGCTGATA CTGTTCAGTG TGGTTCGTTT TGTTAGCTGG CCTTATACTT AGGACCCTAC CGATGTGGAT CTGCAGAAAA TAGAGT

Tri1 upstream region

1001 GACAACACTC AATGAAACGG TCTAACCGTG AGGCAAGCCA TGTCACGGGC CCTGTAGGTA TATGTTATAA ATGTAGGATA CCCTCTACAA CTCT

pTub_S1
Tri1 upstream region
TUB promoter

1101 TATCTCAAAC ACAACTGGTA TCTCTTCCAA GGCTTGACAG ACCATTTCGC TGTCCCCCCG GTGACTCTTG AGGCACATCA AGACCTGTTT GTATGC

dTri1-U_AS

TUB promoter

1201 TAGAATGATT CAGCGCTCTG ATCTGACTGA TACAGCACCA ATTATGCTCA GAGAAAGACA TATTCAATGG AGCCTGTGTT GTAGAGCAGG ATAT

TUB promoter

1301 ATGGCGGCCG TCTAAGGAGC GCACTACCGA TAACGCAGTT ACACAGTTTA TAAGGGCTTT TTGCCAAACA AGGTATGCCT TAGTGGCATG TTAG

TUB promoter

1401 TATTGGGCCT GCCCGACAAG CTGCAACAGA TAACCCCAAC TGTCAGCACG GCATTTTACG AGTAGGTCCT ACCTGCACAT ATCCAAATGA TGAA

TUB promoter

1501 TTACTGTTAC GATAAACTGA TAGATGATGC GCTATTCACA GCGCCCTGAG ATTCCATGGT AGTGTCCCTT TTTTGGGTCC TCAACCATTG TCCTCA

TUB promoter

1601 CCTGACCGTC AAACTGATCT CAAGAGAGGG ACAAAATGCT ACCAGTATGG AGAGTCATTT TCAAGCACCT AGAAAGGTAT GATCGTGCTT GGAC

TUB promoter

1701 TCATATACAG CTGTACTGAA GTATACCTGG CGTGGTTCGC GCAGCAAAGA CCAAAGAGTG GTTCGTTCCT GAGGTCTATG ACTAATCCAG GCGC

TUB promoter

1801 GGACCTGGCG GTTCCCCTTT TGCCACGTGA CATATGTTGT TGTTGATCTT TTTCTCCTTT ACTACGGGCG ATCCACTTAG TTGGCTGGCC TCGCTCT

TUB promoter

1901 CTGGCGGCTT CACCGCTCAA ATTCCCCCAA CCCAACTTTC CTTCTCCTGA TCTCGCTCCT GTCCTCTTCC CTCCTCCCCT CAATTCCGGC TGTTCCC

pTubhph_S

TUB promoter hph

2001 TCTATCTTCA TCCGACTTCG ATTCTTTTTA CGGTTTCTGC AAATAATCCT TTTTTTAATC CTCTATACCC GTCCACACCC TTCACATCGA TATGAAAA

pTub_AS

hph

pTubhph_S

2101 CCTGAACTCA CCGCGACGTC TGTCGAGAAG TTTCTGATCG AAAAGTTCGA CAGCGTCTCC GACCTGATGC AGCTCTCGGA GGGCGAAGAA TCTC

hph

2201 TCAGCTTCGA TGTAGGAGGG CGTGGATATG TCCTGCGGGT AAATAGCTGC GCCGATGGTT TCTACAAAGA TCGTTATGTT TATCGGCACT TTGCA

hph

2301 CGCGCTCCCG ATTCCGGAAG TGCTTGACAT TGGGGAGTTC AGCGAGAGCC TGACCTATTG CATCTCCCGC CGTGCACAGG GTGTCACGTT GCAA

hph

2401 CCTGAAACCG AACTGCCCGC TGTTCTCCAG CCGGTCGCGG AGGCCATGGA TGCGATCGCT GCGGCCGATC TTAGCCAGAC GAGCGGGTTC GGC

hph

2501 GACCGCAAGG AATCGGTCAA TACACTACAT GGCGTGATTT CATATGCGCG ATTGCTGATC CCCATGTGTA TCACTGGCAA ACTGTGATGG ACGA

hph

2601 CAGTGCGTCC GTCGCGCAGG CTCTCGATGA GCTGATGCTT TGGGCCGAGG ACTGCCCCGA AGTCCGGCAC CTCGTGCATG CGGATTTCGG CTCC

hph

2701 GTCCTGACGG ACAATGGCCG CATAACAGCG GTCATTGACT GGAGCGAGGC GATGTTCGGG GATTCCCAAT ACGAGGTCGC CAACATCCTC TTC

hph

2801 CGTGGTTGGC TTGTATGGAG CAGCAGACGC GCTACTTCGA GCGGAGGCAT CCGGAGCTTG CAGGATCGCC GCGCCTCCGG GCGTATATGC TCC

hph

2901 TCTTGACCAA CTCTATCAGA GCTTGGTTGA CGGCAATTTC GATGATGCAG CTTGGGCGCA GGGTCGATGC GACGCAATCG TCCGATCCGG AGCC

hph

3001 GTCGGGCGTA CACAAATCGC CCGCAGAAGC GCGGCCGTCT GGACCGATGG CTGTGTAGAA GTACTCGCCG ATAGTGGAAA CCGACGCCCC AG

hph

HSVtk

3101 CGAGGGCAAA GGAAATGGCT TCGTACCCCG GCCATCAACA CGCGTCTGCG TTCGACCAGG CTGCGCGTTC TCGCGGCCAT AGCAACCGAC GTA

HSVtk

3201 GCGCCCTCGC CGGCAGCAAG AAGCCACGGA AGTCCGCCCG GAGCAGAAAA TGCCCACGCT ACTGCGGGTT TATATAGACG GTCCCCACGG GA

HSVtk

3301 ACCACCACCA CGCAACTGCT GGTGGCCCTG GGTTCGCGCG ACGATATCGT CTACGTACCC GAGCCGATGA CTTACTGGCG GGTGCTGGGG GCT

HSVtk

3401 CAATCGCGAA CATCTACACC ACACAACACC GCCTCGACCA GGGTGAGATA TCGGCCGGGG ACGCGGCGGT GGTAATGACA AGCGCCCAGA TA

HSVtk

3501 CATGCCTTAT GCCGTGACCG ACGCCGTTCT GGCTCCTCAT ATCGGGGGGG AGGCTGGGAG CTCACATGCC CCGCCCCCGG CCCTCACCCT CATC

HSVtk

3601 CGCCATCCCA TCGCCGCCCT CCTGTGCTAC CCGGCCGCGC GGTACCTTAT GGGCAGCATG ACCCCCCAGG CCGTGCTGGC GTTCGTGGCC CTCA

HSVtk

3701 CGACCTTGCC CGGCACCAAC ATCGTGCTTG GGGCCCTTCC GGAGGACAGA CACATCGACC GCCTGGCCAA ACGCCAGCGC CCCGGCGAGC GG

HSVtk

3801 GGCTATGCTG GCTGCGATTC GCCGCGTTTA CGGGCTACTT GCCAATACGG TGCGGTATCT GCAGTGCGGC GGGTCGTGGC GGGAGGACTG GGG

HSVtk

3901 TCGGGGACGG CCGTGCCGCC CCAGGGTGCC GAGCCCCAGA GCAACGCGGG CCCACGACCC CATATCGGGG ACACGTTATT TACCCTGTTT CGG

HSVtk

4001 AGTTGCTGGC CCCCAACGGC GACCTGTATA ACGTGTTTGC CTGGGCCTTG GACGTCTTGG CCAAACGCCT CCGTTCCATG CACGTCTTTA TCCTG

HSVtk

4101 CGACCAATCG CCCGCCGGCT GCCGGGACGC CCTGCTGCAA CTTACCTCCG GGATGGTCCA GACCCACGTC ACCACCCCCG GCTCCATACC GAC

dTri1-D_S

HSVtk Tri1 downstream region

4201 GACCTGGCGC GCACGTTTGC CCGGGAGATG GGGGAGGCTA ACTGACGTAG GAGGACGTCA CAGTCTTGGT CAGATCATCG TTATGCTGTA TTTC

hphtkC2_AS

Tri1 downstream region

4301 ACGACGTTTG CCTCGAGTGG AGGACCATCC TTGGGTTGAC CGGCGAAGTA AATGGGAAGT CATATTTCTT TTGTAGTCGT TGTTATAGAT CGCTG

Tri1 downstream region

4401 CGGTTTACAT AGTTGGGTTT GATCTCGCCA CTTGTTGACG GTAACATCTC GCCCAAAAAG CCCAATGAAA ATAAATTATT GTTTATTAGT CGAATC

Tri1 downstream region

4501 TCTGTATATG AATCTTTTTT CAACATGGGA ATATCGAACT CAGAATTCAA ATGCAAGCAT TCGCACCTTG TTTGCATTGG CAGCACGTTG AATCAT

Tri1 downstream region

4601 AGTTCGAATT ACCCAGTTGA TGCAACAGTG AAAGAAAACT TGGTTGTCGA ATAAAAATAT ACTTGGGGGT ACCGAGCTCG AATTCGTAAT CATG

dTri1-D_AS

4701 GCTGTTTCCT GTGTGAAATT GTTATCCGCT CACAATTCCA CACAACATAC GAGCCGGAAG CATAAAGTGT AAAGCCTGGG GTGCCTAATG AGTGAGCTAA

4801 CTCACATTAA TTGCGTTGCG CTCACTGCCC GCTTTCCAGT CGGGAAACCT GTCGTGCCAG CTGCATTAAT GAATCGGCCA ACGCGCGGGG AGAGGCGGTT

4901 TGCGTATTGG GCGCTCTTCC GCTTCCTCGC TCACTGACTC GCTGCGCTCG GTCGTTCGGC TGCGGCGAGC GGTATCAGCT CACTCAAAGG CGGTAATACG

5001 GTTATCCACA GAATCAGGGG ATAACGCAGG AAAGAACATG TGAGCAAAAG GCCAGCAAAA GGCCAGGAAC CGTAAAAAGG CCGCGTTGCT GGCGTTTTTC

5101 CATAGGCTCC GCCCCCCTGA CGAGCATCAC AAAAATCGAC GCTCAAGTCA GAGGTGGCGA AACCCGACAG GACTATAAAG ATACCAGGCG TTTCCCCCTG

5201 GAAGCTCCCT CGTGCGCTCT CCTGTTCCGA CCCTGCCGCT TACCGGATAC CTGTCCGCCT TTCTCCCTTC GGGAAGCGTG GCGCTTTCTC ATAGCTCACG

5301 CTGTAGGTAT CTCAGTTCGG TGTAGGTCGT TCGCTCCAAG CTGGGCTGTG TGCACGAACC CCCCGTTCAG CCCGACCGCT GCGCCTTATC CGGTAACTAT

5401 CGTCTTGAGT CCAACCCGGT AAGACACGAC TTATCGCCAC TGGCAGCAGC CACTGGTAAC AGGATTAGCA GAGCGAGGTA TGTAGGCGGT GCTACAGAGT

5501 TCTTGAAGTG GTGGCCTAAC TACGGCTACA CTAGAAGGAC AGTATTTGGT ATCTGCGCTC TGCTGAAGCC AGTTACCTTC GGAAAAAGAG TTGGTAGCTC

5601 TTGATCCGGC AAACAAACCA CCGCTGGTAG CGGTGGTTTT TTTGTTTGCA AGCAGCAGAT TACGCGCAGA AAAAAAGGAT CTCAAGAAGA TCCTTTGATC

5701 TTTTCTACGG GGTCTGACGC TCAGTGGAAC GAAAACTCAC GTTAAGGGAT TTTGGTCATG AGATTATCAA AAAGGATCTT CACCTAGATC CTTTTAAATT

5801 AAAAATGAAG TTTTAAATCA ATCTAAAGTA TATATGAGTA AACTTGGTCT GACAGTTACC AATGCTTAAT CAGTGAGGCA CCTATCTCAG CGATCTGTCT

bla

5901 ATTTCGTTCA TCCATAGTTG CCTGACTCCC CGTCGTGTAG ATAACTACGA TACGGGAGGG CTTACCATCT GGCCCCAGTG CTGCAATGAT ACCGCGAGAC

bla

6001 CCACGCTCAC CGGCTCCAGA TTTATCAGCA ATAAACCAGC CAGCCGGAAG GGCCGAGCGC AGAAGTGGTC CTGCAACTTT ATCCGCCTCC ATCCAGTCTA

bla

6101 TTAATTGTTG CCGGGAAGCT AGAGTAAGTA GTTCGCCAGT TAATAGTTTG CGCAACGTTG TTGCCATTGC TACAGGCATC GTGGTGTCAC GCTCG

bla

6201 TGGTATGGCT TCATTCAGCT CCGGTTCCCA ACGATCAAGG CGAGTTACAT GATCCCCCAT GTTGTGCAAA AAAGCGGTTA GCTCCTTCGG TCCTCCGATC

bla

6301 GTTGTCAGAA GTAAGTTGGC CGCAGTGTTA TCACTCATGG TTATGGCAGC ACTGCATAAT TCTCTTACTG TCATGCCATC CGTAAGATGC TTTTCTGTGA

bla

6401 CTGGTGAGTA CTCAACCAAG TCATTCTGAG AATAGTGTAT GCGGCGACCG AGTTGCTCTT GCCCGGCGTC AATACGGGAT AATACCGCGC CACATAGCAG

bla

6501 AACTTTAAAA GTGCTCATCA TTGGAAAACG TTCTTCGGGG CGAAAACTCT CAAGGATCTT ACCGCTGTTG AGATCCAGTT CGATGTAACC CACTCGTGCA

bla

6601 CCCAACTGAT CTTCAGCATC TTTTACTTTC ACCAGCGTTT CTGGGTGAGC AAAAACAGGA AGGCAAAATG CCGCAAAAAA GGGAATAAGG GCGACACGGA

bla

6701 AATGTTGAAT ACTCATACTC TTCCTTTTTC AATATTATTG AAGCATTTAT CAGGGTTATT GTCTCATGAG CGGATACATA TTTGAATGTA TTTAGAAAAA

bla

6801 TAAACAAATA GGGGTTCCGC GCACATTTCC CCGAAAAGTG CCACCTGACG TCTAAGAAAC CATTATTATC ATGACATTAA CCTATAAAAA TAGGCGTATC

6901 ACGAGGCCCT TTCGTCTCGC GCGTTTCGGT GATGACGGTG AAAACCTCTG ACACATGCAG CTCCCGGAGA CGGTCACAGC TTGTCTGTAA GCGGATGCCG

7001 GGAGCAGACA AGCCCGTCAG GGCGCGTCAG CGGGTGTTGG CGGGTGTCGG GGCTGGCTTA ACTATGCGGC ATCAGAGCAG ATTGTACTGA GAGTGCACCA

7101 TATGCGGTGT GAAATACCGC ACAGATGCGT AAGGAGAAAA TACCGCATCA GGCGCCATTC GCCATTCAGG CTGCGCAACT GTTGGGAAGG GCGATCGGTG

7201 CGGGCCTCTT CGCTATTACG CCAGCTGGCG AAAGGGGGAT GTGCTGCAAG GCGATTAAGT T

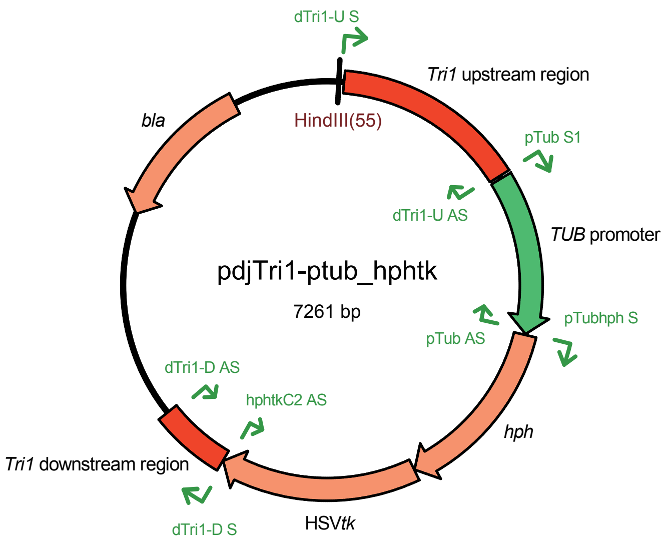

Supplementary Figure S7. Sequences and maps of replacement and disruption vectors. Gene replacement vectors pCjcTri3_V467G and pCjH3Tri8 were used for transformation of transgenic strains YN_153 (*Tri3* locus: *hph::tk*) and YN_145 (*Tri8* locus: *hph::tk*), respectively, to generate marker-free strains EK_001 and YN_155 (**Supplementary Figure S1**). Gene disruption vector pdjTri1-ptub_hphtk was used for transformation of YN_155 to generate YN_173 (*Tri1* locus: *hph::tk*) (**Supplementary Figure S1**).

References

1. Laurence, M. H.; Summerell, B. A.; Burgess, L. W.; Liew, E. C. Y., *Fusarium burgessii* sp. nov. representing a novel lineage in the genus *Fusarium*. *Fungal Divers* **2011,** *49*, 101-112.
2. O’Donnell, K.; McCormick, S. P.; Busman, M.; Proctor, R. H.; Ward, T. J.; Doehring, G.; Geiser, D. M.; Alberts, J. F.; Rheeder, J. P., Marasas et al. 1984 “Toxigenic *Fusarium* species: identity and mycotoxicology” revisited. *Mycologia* **2018,** *110*, 1058-1080.
3. O’Donnell, K.; Rooney, A. P.; Proctor, R. H.; Brown, D. W.; McCormick, S. P.; Ward, T. J.; Frandsen, R. J. N.; Lysøe, E.; Rehner, S. A.; Aoki, T.; Robert, V. A. R. G.; Crous, P. W.; Groenewald, J. Z.; Kang, S.; Geiser, D. M., Phylogenetic analyses of *RPB1* and *RPB2* support a middle Cretaceous origin for a clade comprising all agriculturally and medically important fusaria. *Fungal Genet Biol* **2013,** *52*, 20-31.
4. Yilmaz, N.; Sandoval-Denis, M.; Lombard, L.; Visagie, C. M.; Wingfield, B. D.; Crous, P. W., Redefining species limits in the *Fusarium fujikuroi* species complex. *Persoonia* **2021,** *46*, 129-162.
5. O'Donnell, K.; Sarver, B., A. J.; Brandt, M.; Chang, D., C.; Noble-Wang, J.; Park, B., J.; Sutton, D., A.; Benjamin, L.; Lindsley, M.; Padhye, A.; Geiser, D., M.; Ward, T., J., Phylogenetic diversity and microsphere array-based genotyping of human pathogenic fusaria, Including Isolates from the multistate contact lens-associated U.S. keratitis outbreaks of 2005 and 2006. *J Clin Microbiol* **2007,** *45*, 2235-2248.
6. Koizumi, Y.; Nakajima, Y.; Tanaka, Y.; Matsui, K.; Sakabe, M.; Maeda, K.; Sato, M.; Koshino, H.; Sato, S.; Kimura, M.; Takahashi-Ando, N., A role in 15-deacetylcalonectrin acetylation in the non-enzymatic cyclization of an earlier bicyclic intermediate in *Fusarium* trichothecene biosynthesis. *Int J Mol Sci* **2024,** *25*, 4288.
7. Nakajima, Y.; Tanaka, Y.; Matsui, K.; Maeda, K.; Kitou, Y.; Kanamaru, K.; Ohsato, S.; Kobayashi, T.; Takahashi-Ando, N.; Kimura, M., Accumulation of an unusual trichothecene shunt metabolite in liquid culture of *Fusarium*

graminearum with methionine as the sole nitrogen source. *JSM Mycotoxins* **2017,** *67*, 7-9.

8. Takahashi-Ando, N.; Tokai, T.; Yoshida, M.; Fujimura, M.; Kimura, M., An easy method to identify 8-keto-15-hydroxytrichothecenes by thin-layer chromatography. *Mycotoxins* **2008,** *58*, 115-117.
